# Long Non-Coding RNAs in Multiple Sclerosis—Differential Expression and Functional Implications

**DOI:** 10.3390/genes16111327

**Published:** 2025-11-03

**Authors:** Kaalindi Misra, Aishwary Nerkar, Ferdinando Clarelli, Melissa Sorosina, Federica Esposito

**Affiliations:** 1 Laboratory of Human Genetics of Neurological Disorders, Division of Neuroscience, IRCCS San Raffaele Scientific Institute, 20132 Milan, Italynerkar.aishwary@hsr.it (A.N.); sorosina.melissa@hsr.it (M.S.); 2 Neurology and Neurorehabilitation Unit, IRCCS San Raffaele Scientific Institute, 20132 Milan, Italy

**Keywords:** MS, RRMS, SPMS, CNS, lncRNA

## Abstract

Background/Objectives: Long non-coding RNAs (lncRNAs) are increasingly recognized as key regulators of immune pathways and may hold diagnostic and therapeutic relevance in autoimmune diseases such as Multiple Sclerosis (MS). However, research on lncRNAs in MS remains fragmented and geographically clustered. This systematic review aimed to collate and critically evaluate studies of lncRNA expression in MS, assess consistency of findings across studies, and synthesize proposed functional implications of the most frequently studied lncRNAs. Methods: This PROSPERO-registered review (CRD420250575938), conducted in accordance with PRISMA, searched PubMed, Scopus, Embase, and Web of Science (2010–2024) for studies evaluating lncRNA expression in adult MS (≥18 years of age). Eligible studies included ≥20 participants and assessed lncRNAs in blood, PBMCs, serum, plasma, or CSF using qRT-PCR, RNA-seq, or microarrays. Pediatric, review, animal, and in vitro studies were excluded. Two reviewers independently screened and extracted data, with risk of bias evaluated using QUADAS-2. Results: Narrative synthesis of 51 studies identified 77 unique lncRNAs. A limited set (MALAT1, GAS5, MEG3, H19) demonstrated consistent dysregulation in MS, whereas others (THRIL, IFNG-AS1, HOTAIR, TUG1) exhibited context-dependent expression influenced by treatment, relapse status, or demographics. Functional annotations converged on immune pathways, including NF-κB, STAT3, IFN-γ/Th1, and glucocorticoid signaling. Conclusions: This review identifies reproducible and context-specific lncRNA dysregulation in MS, emphasizing the need for transcriptome-wide approaches, standardized methods, and multi-center validation. Current evidence is constrained by geographic clustering, preselection bias, and methodological heterogeneity.

## 1. Introduction

Multiple sclerosis (MS) is a chronic autoimmune disorder of the central nervous system (CNS) that causes inflammation, demyelination, and neurodegeneration [1]. The exact cause of MS is unknown, but a combination of genetic, environmental, and immune-related factors plays a significant role in the disease’s onset and progression [2]. With the advent of genomics, long non-coding RNAs (lncRNAs), a subclass of non-coding RNA molecules, have been associated with MS pathology. They can act as molecular scaffolds to shape chromatin architecture, decoys for transcription factors, sponges for microRNAs, or guides for chromatin-modifying complexes. Like other non-coding RNA species, lncRNAs are generally expressed at low levels, display high cell and tissue specificity, and remain largely uncharacterized, with only a minority of the thousands identified having been functionally annotated to date [3]. These unique features make them both challenging to study and potentially valuable as biomarkers or therapeutic targets. In the context of MS, their roles in immune regulation, inflammatory signaling, and neurodegeneration are beginning to be elucidated.

Several lncRNAs have been associated with MS. For instance, vitamin D receptor-associated lncRNAs, including SNHG6, SNHG16, and LINC00346, were significantly down-regulated in MS patients compared to controls, while FLICR, a T cell-related lncRNA, showed potential as a biomarker in a separate cohort [4,5]. Additionally, IFNG-AS1 lncRNA exhibited differential expression in MS patients, showing a positive correlation with disease duration and a negative correlation with age at onset [4]. Other lncRNAs, including MIAT, H19, NRON, NEAT1, and KCNQ1OT1, exhibited differential expression, with NEAT1 and MALAT1 contributing to Th1/Th2 imbalance in MS through interactions with microRNAs miR-210-3p and miR-544a [6,7]. GAS5 lncRNA was linked to MS severity as it positively correlates with the expanded disability status scale (EDSS) scores in the relapsing–remitting MS patients (RRMS) [8]. Considering all this evidence, it appears that dysregulated lncRNA expression could play a role in modulating critical disease mechanisms in MS, including immune cell function, inflammatory responses, and damage to the CNS.

Thus, it is essential to consolidate and review the existing research on lncRNAs in MS and explore their functional implications. The primary aim of this study is to identify the lncRNAs that are implicated in MS and analyze their differential expression patterns. The secondary aim is to elucidate the functional implications of these lncRNAs in the pathogenesis of MS, providing insights into their roles in disease mechanisms and potential therapeutic applications.

## 2. Materials and Methods

**Figure 1:**
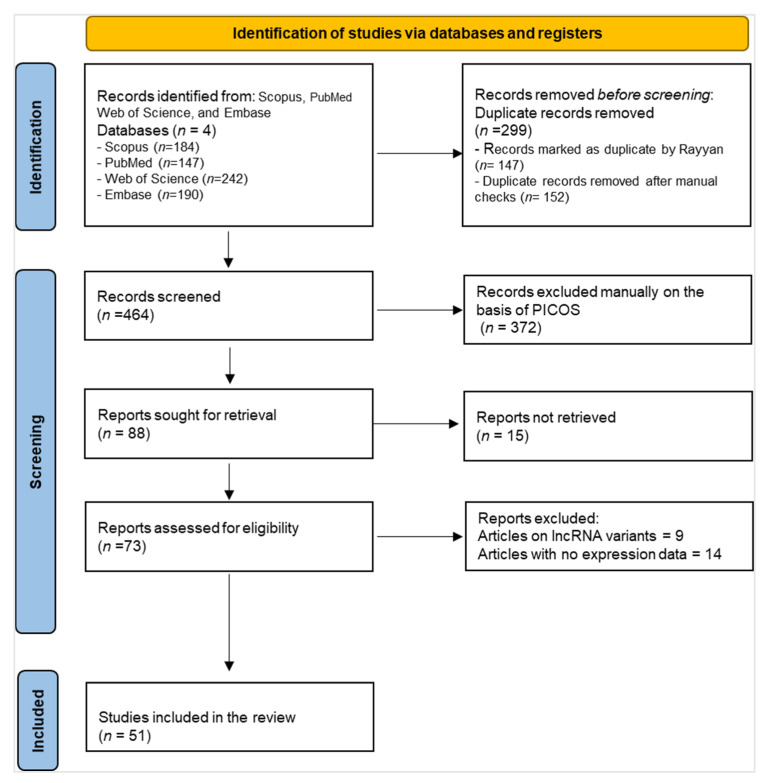
PRISMA flow diagram of the article selection process [9,11]. In this chart, “*n*” represents the number for that box.

Following the Preferred Reporting Items for Systematic Review and Meta-analysis (PRISMA) recommendations, this systematic review was designed and executed as shown in Figure 1 [9]. The review protocol was registered in PROSPERO (#CRD420250575938), with all methodological details and eligibility parameters established beforehand [10].

### 2.1. Search Strategy and Inclusion/Exclusion Criteria

Literature was searched in the following databases: PubMed, Scopus, Embase and Web of Science using combinations of the following keywords: “long non-coding RNAs,” “lncRNAs,” “multiple sclerosis,” “autoimmune diseases,” “gene regulation,” and “differential expression.” Only peer-reviewed original research articles published in English from January 2010 to August 2024 were included. Searches were last updated on 25 August 2024. This interval was selected to capture the rise in research focused on the roles of lncRNAs outside of oncology, a trend that became prominent in the early 2010s. Studies that focused on lncRNAs in human MS patients were considered. Exclusion criteria were review articles, case reports, and studies not involving MS. Data extraction focused on lncRNA expression patterns, methods used for identification, and functional analyses.

Criteria for study selection were formulated in accordance with the Population, Intervention, Comparison, Outcomes and Study design (PICOS) framework as presented in Table 1 [12]. Only original research studies were eligible for inclusion. Review articles were excluded, but reviewed to inform the overall context of findings. For the intervention domain, only studies assessing lncRNA expression in whole blood, PBMCs, serum, plasma or cerebrospinal fluid (CSF) were included. This decision was made because (i) both matrices are clinically accessible and commonly used for biomarker discovery in MS, (ii) they directly reflect systemic or CNS-related immune and inflammatory activity, and (iii) the majority of MS transcriptomic work in clinical cohorts has utilized molecular techniques such as qRT-PCR, RNA sequencing, or microarray profiling in these tissues, facilitating comparability across studies. For the population, studies were required to include adult patients (≥18 years), since pediatric MS has specific clinical features which could introduce heterogeneity. Furthermore, a minimum sample size of 20 subjects in total was set as a threshold to reduce the risk of inflated effect sizes and unstable variance estimates commonly associated with very small cohorts. This cutoff is consistent with methodological standards in biomarker discovery studies, where extremely small sample sizes (<20) are often considered exploratory and insufficient for meaningful inference.

### 2.2. Study Selection, Data Extraction and Analysis

Titles and abstracts were independently screened by two reviewers (K.M. and A.N.) to identify studies meeting the eligibility criteria. Discrepancies regarding the inclusion of the articles were resolved through discussion with a third independent reviewer (M.S). For all eligible studies, data were extracted on the following variables: first author, year of publication, study design (cross-sectional or longitudinal), sample size per group, exclusion criteria for subject enrollment, proportion of females, mean age, average disease duration, disease course, functional scale scores specific for each disorder, biological source of analysis, analytical technique, and validation methods. Data extraction was conducted independently and in duplicate to ensure accuracy. Due to substantial methodological and reporting heterogeneity, quantitative synthesis and meta-analysis were not performed; instead, results were summarized narratively, organized by lncRNA, biological source, and clinical context.

### 2.3. Risk of Bias Assessment

The methodological quality and risk of bias of each included study were evaluated using the Quality Assessment for Diagnostic Accuracy Studies-2 (QUADAS-2) tool by two independent reviewers (K.M. and A.N.) [13]. A domain was classified as having a “high risk of bias” if at least one key question within that domain received a negative response, and as “unclear” if any question was judged to have insufficient information. Disagreements were resolved through consultation with a third reviewer (M.S.). Although no formal statistical approach was used to detect reporting bias, potential sources of bias were qualitatively noted, particularly in cases where studies originated from overlapping geographic regions, institutes, or research groups. The overall certainty of the body of evidence was not formally graded.

## 3. Results

### 3.1. Study Selection and Characteristics

In total, 763 studies were retrieved from PubMed, Scopus, Web of Science, and Embase. All retrieved articles were uploaded into Rayyan software (Qatar Computing Research Institute, Doha, Qatar) for systematic review management [11]. The platform was used to automatically identify and remove duplicate records, and to facilitate blinded screening of titles and abstracts by the review authors. After deduplication, 464 studies were screened for title and abstract reading, with eighty-eight studies undergoing full-text review. Consequently, fifty-one studies met the inclusion criteria as detailed in Figure 1 and were included in this systematic review as detailed in Table S1.

**Figure 2:**
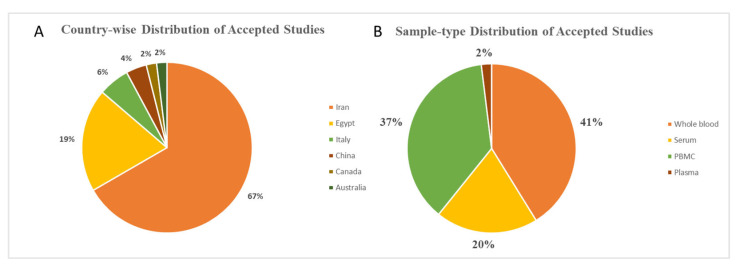
(**A**). Pie chart of the country of origin of the accepted studies. 67% of the studies are from Iran, followed by Egypt with 19%, Italy with 6%, China with 4%, and Canada and Australia with 2% each. (**B**). Pie chart of sample type distribution of the accepted studies. 41% of the studies explored whole blood, followed by 37% studying PBMCs, 20% of studies conducted on serum and 2% on plasma.

Most studies were case–control in design (70%), and the majority (67%) were conducted in Iran, indicating potential geographic clustering. The primary assay technique used was RT-qPCR (84%), with a few studies employing microarrays (10%) or RNA sequencing (6%). Risk of bias was assessed using the QUADAS-2 tool. Of the 51 included studies, 47 were rated low risk, 2 had high risk, and 2 had unclear risk, primarily due to issues in patient selection. Overall, study quality was acceptable, with low concern for bias in most domains. Most studies focused on RRMS, followed by secondary progressive MS (SPMS) and primary progressive MS (PPMS). Tissue types varied: 41% used peripheral whole blood, 37% used PBMCs, 20% used serum, and 2% used plasma. No included studies used CSF. Tissue type distribution is shown in Figure 2. While the included studies varied in tissue type and location, there was notable consistency in the selection of specific lncRNAs for analysis. This trend is explored in detail in the following section. Most studies (43/51) focused on RRMS cohorts, while only a minority investigated progressive subtypes (SPMS, PPMS). MALAT1, MEG3 and GAS5 were primarily studied in RRMS populations, whereas evidence in progressive MS is sparse, limiting conclusions about subtype-specific dysregulation.

### 3.2. lncRNA Frequency and Trends

**Figure 3:**
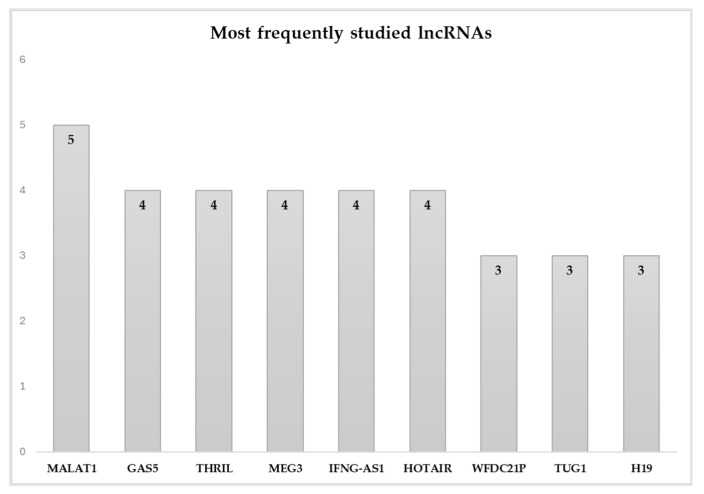
Bar plot of the frequency of studied lncRNAs in the accepted studies.

Across the 51 included studies, a total of 77 unique lncRNAs were investigated. However, a clear trend toward the repeated analysis of specific lncRNAs selected according to a priori hypothesis was observed, indicating a strong pre-selection bias in the literature. As shown in Figure 3, MALAT1 stands out as the most frequently studied lncRNA, appearing in five independent studies. This was followed by a group of lncRNAs, GAS5, THRIL, MEG3, IFNG-AS1, and HOTAIR, each reported in four studies. Additional recurrent lncRNAs include WFDC21P, TUG1, and H19, each featured in three publications. The full frequency distribution is summarized in Tables S1 and S2 and demonstrated in Figure 3.

This pattern reveals that the field is currently dominated by a narrow subset of well-known lncRNAs, many of which have established regulatory roles in inflammation, apoptosis, or immune signaling. The frequent appearance of these lncRNAs across multiple studies suggests a hypothesis-driven rather than discovery-driven approach to biomarker identification. While this targeted strategy is expected, given that a large proportion of lncRNA are currently functionally uncharacterized, and offers some mechanistic depth, it may limit the identification of novel, unbiased diagnostic or prognostic biomarkers for MS.

It is important to note that some lncRNAs frequently reported in the literature have undergone reclassification by the HUGO Gene Nomenclature Committee databases [14]. For instance, TUG1, previously considered as lncRNA (alias: LINC00080), is now classified as a protein-coding gene in the HGNC database. Similarly, RMRP, which was studied as a lncRNA in several papers, is now listed as a miscellaneous RNA (RNA, misc)—a category for non-protein-coding RNAs with diverse functions. However, alternate resources such as GeneCards.org continue to classify RMRP as a lncRNA, reflecting discrepancies in gene annotation across databases. These evolving annotations underscore the need for standardized classification frameworks and consistent database referencing in future transcriptomic studies. Misclassification or outdated annotations may lead to the inclusion of genes that no longer meet the current criteria for lncRNAs, potentially skewing conclusions and limiting reproducibility.

### 3.3. Risk of Bias

**Figure 4:**
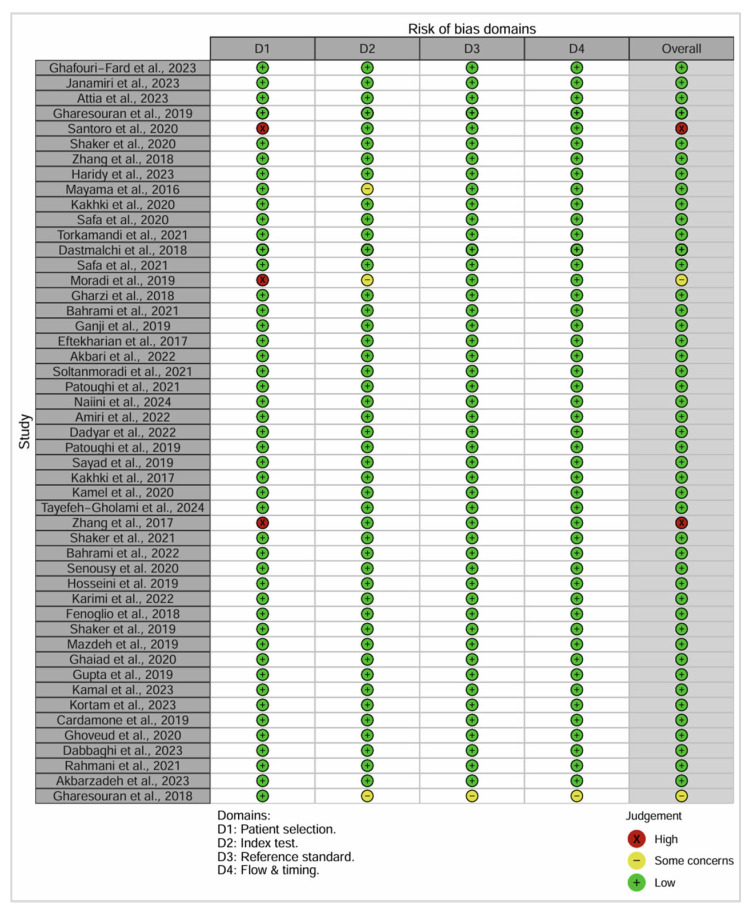
Traffic plot of the included studies of lncRNAs after application of QUADAS-2 assessment [4,5,7,8,13,15,16,17,18,19,20,21,22,23,24,25,26,27,28,29,30,31,32,33,34,35,36,37,38,39,40,41,42,43,44,45,46,47,48,49,50,51,52,53,54,55,56,57,58,59].

**Figure 5:**
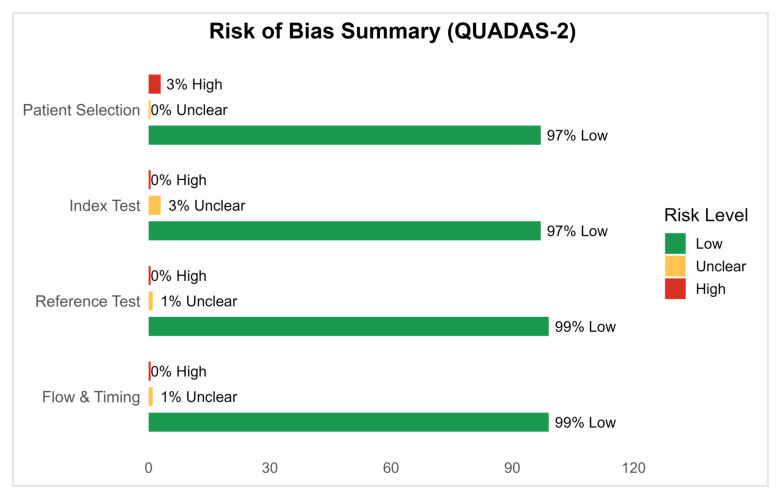
QUADAS-2 risk of bias summary across all included studies [13].

Risk of bias across the 51 included studies was assessed using the QUADAS-2 tool, which evaluates four domains: patient selection, index test, reference standard, and flow and timing [13]. The results are summarized in Figure 4 (traffic light plot) and Figure 5 (risk of bias summary).

Most of the studies (46/51) were assessed as low bias across most domains. Two studies showed a high risk of bias, primarily due to non-randomized patient selection or lack of blinding. Additional two studies were rated as having unclear risk, typically due to insufficient detail on sample handling or outcome measurement. The index test domain generally scored well, as most studies clearly described the lncRNA quantification methods.

However, applicability concerns were identified in a subset of studies, particularly those that evaluated lncRNAs that are now reclassified or ambiguously annotated. Especially, TUG1, which was studied as a lncRNA in three papers, is now classified as a protein-coding gene according to the HGNC database (HGNC: 26066). Similarly, RMRP has been reassigned to the miscellaneous RNA (HGNC: 10031) category.

These reclassifications raise concerns about classification and applicability bias, as the studies in question may have drawn diagnostic or mechanistic conclusions under outdated assumptions about gene function. This highlights the importance of referencing up-to-date gene annotations during study design and systematic review. Considering this, the inclusion of such studies was noted as a limitation in overall risk assessment, although their methodological conduct was otherwise sound. The impact of this risk of bias on the overall interpretation of diagnostic value is further addressed in the Discussion section.

### 3.4. Observed Differential Expression of Most Studied lncRNAs

Several lncRNAs were evaluated in three or more independent studies, as shown in Figure 3. The genes highlighted in Figure 3 were selected for examination, representing the most frequently studied lncRNAs with reproducible evidence of differential expression across MS studies. We examined their reported direction of expression to assess consistency and possible functional implications of lncRNAs in core disease-related expression changes (MS vs. healthy controls, HCs) and context-specific modulations observed under particular treatments or clinical conditions. To improve clarity and interpretability, we divided the summary of lncRNA findings into two complementary tables. Table 2 presents results restricted to the contrast between MS patients and HCs, enabling a consistent evaluation of directionality (up- or downregulation in MS) and assessment of replicability across studies. Functional implications are summarized from the primary studies, with emphasis on immune modulation, inflammatory signaling, and MS-related pathways. Table 3, in contrast, collates findings from studies that examined lncRNA expression in other clinical contexts, such as treatment response (Disease Modifying Therapies (DMTs), vitamin D supplementation), relapse status, disease course (RRMS vs. SPMS), and demographic subgroups (sex, ethnicity).

Several lncRNAs (THRIL, HOTAIR, TUG1, H19) show upregulation in active or progressive MS states, particularly in SPMS or relapse phases, linking them to inflammatory and immune signaling pathways (e.g., STAT3, NF-κB, TNF-α). Ethnic, sex, and treatment-specific patterns (THRIL, H19, MEG3) suggest context-dependent regulation. HOTAIR and H19, in particular, emerge as promising biomarkers for relapse and progression.

**Figure 6:**
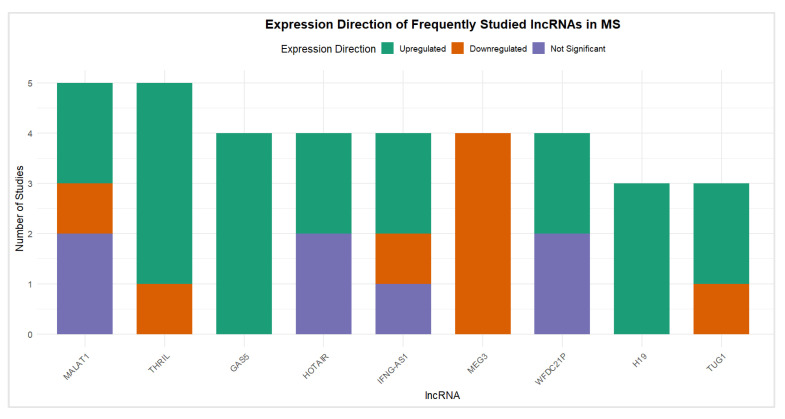
Vote-counting summary of expression direction for frequently studied lncRNAs in MS. The bar plot illustrates the number of studies reporting each lncRNA as upregulated, downregulated, or not significantly changed across MS cohorts.

To assess the consistency of expression trends among the most frequently investigated lncRNAs, a vote-counting bar plot was constructed (Figure 6) to depict the proportion of studies reporting upregulation. GAS5 and H19 emerged as the most consistently upregulated lncRNAs, whereas MEG3 was consistently downregulated, each reported in ≥75% of studies, based on data summarized in Tables S1 and S2 [28,32,38,39,40,41,42,43,44,45,46,47,48,49,50,51,52,53,54,55,56,57,58,59,60,61]. Most lncRNAs, including MALAT1, GAS5, THRIL, HOTAIR, and H19, show predominant upregulation, indicating their consistent association with pro-inflammatory or disease-activity–related mechanisms.

## 4. Discussion

Long non-coding RNAs have emerged as key regulatory molecules implicated in immune modulation and neuroinflammation, both central to the pathogenesis of MS. Through this systematic review, we evaluated the current body of evidence surrounding lncRNA expression profiles in MS patients, assessing both their reproducibility and functional relevance. The findings provide insight into commonly studied candidates, methodological patterns, and important limitations in the field. Below, we synthesize the key outcomes, highlight critical gaps, and suggest future directions.

### 4.1. Summary of Key Findings

This systematic review analyzed 51 eligible studies investigating lncRNAs in MS, covering a total of 77 unique lncRNAs. Among these, a small subset of lncRNAs were disproportionately studied, particularly MALAT1 (5 studies), GAS5, THRIL, MEG3, IFNG-AS1 (NEST), and HOTAIR (each in 4 studies). Consistency in expression patterns varied: GAS5 and MEG3 showed uniform up- and downregulation, respectively, while MALAT1, THRIL, and HOTAIR displayed partial or conflicting results. Most studies focused on RRMS, with some including SPMS or PPMS cases. Biological sample sources have been systematically summarized in Table S2; the majority of studies utilized PBMCs, followed by serum, whole blood, and plasma. As several frequently studied lncRNAs were assessed across more than one biological source, PBMCs appear to provide a more representative profile of immune-related gene regulation. While expression was typically compared against HC; in a few cases, the analyses were performed stratifying subjects according to treatment status or disease course, as reported in Table 3.

Geographically, a striking concentration was observed: Iran alone accounted for over 60% of the studies, and a significant portion of those came from closely affiliated institutions or recurring author groups, indicating possible clustering of research outputs. This raises concerns about population overlap, as several studies from the same country may have used the same or partially overlapping patient cohorts.

### 4.2. Functional Implication of the Frequently Studied lncRNAs

A major outcome of this review was the identification of a subset of lncRNAs that were not only frequently studied but also demonstrated reproducible expression patterns in MS and biologically relevant functional roles. These lncRNAs, especially MALAT1, GAS5, THRIL, MEG3, IFNG-AS1, HOTAIR, WFDC21P, TUG1, and H19, appear to converge on several immunological and transcriptional pathways central to MS pathophysiology.

Among them, GAS5 and MEG3 exhibited the most consistent expression patterns, serving as putative anti-inflammatory regulators. GAS5, upregulated across all studies, functions as a glucocorticoid receptor decoy and may shape glucocorticoid sensitivity and inflammatory tone. MEG3 was uniformly downregulated in untreated MS, but importantly, its modulation under DMTs (notably fingolimod-induced downregulation in females) underscores drug- and sex-specific regulatory influences. MALAT1, while variably expressed, regulates alternative splicing of MS-relevant genes and may suppress pro-inflammatory macrophage and T-cell profiles. THRIL, generally upregulated, was found to differ across ethnic groups (↑ in Kurdish vs. ↓ in Sistani patients), suggesting gene–environment interactions. H19 showed consistent upregulation and was further linked to relapse, SPMS progression, and female sex, highlighting its potential as a biomarker of disease stage and sex-specific susceptibility. WFDC21P also emerged as upregulated, particularly in female RRMS, supporting its involvement in dendritic cell–mediated immune regulation. By contrast, HOTAIR and TUG1 displayed more context-dependent expression: HOTAIR upregulation associated with relapse and vitamin D deficiency but decreased after supplementation, whereas TUG1 showed discordant patterns (downregulated in some cohorts but upregulated in SPMS), with inverse correlations to disease duration in females, supporting a nuanced, stage- and sex-dependent role.

Collectively, these findings suggest that while a subset of lncRNAs (e.g., GAS5, MEG3, THRIL, IFNG-AS1, H19) demonstrate reproducible links to immune pathways, others (e.g., MALAT1, HOTAIR, TUG1, WFDC21P) reflect more context-, treatment-, or sex-specific regulation. This highlights both their biomarker potential and the need for mechanistic and population-diverse studies to delineate when and how these lncRNAs exert functional influence in MS.

### 4.3. Biases and Limitations in the Literature

One of the most apparent issues in the literature is preselection bias. Most lncRNAs analyzed in these studies were not discovered de novo but were selected based on prior known associations with cancer, immune pathways, or other diseases. Only a handful of studies used high-throughput screening approaches, allowing novel lncRNA discovery. Current evidence for country- or region-specific effects on lncRNA expression in MS is limited. A minority of studies reported population- or ethnicity-specific differences (for example, divergent THRIL expression between Kurdish and Sistani cohorts), suggesting that genetic background or gene–environment interactions could modify lncRNA regulation.

There is also a pronounced geographic bias, not only in the number of studies emerging from a few countries but in the lack of representation from large MS cohorts in Europe, North America, and East Asia, despite the known global distribution of the disease. Approximately 86% of the included studies originated from the Middle East and North Africa (MENA) region, primarily Iran and Egypt. This concentration likely reflects strong regional research interest, accessible clinical populations, and established qRT-PCR-based workflows, rather than a true regional biological specificity. Nevertheless, such clustering can limit the generalizability of findings and introduce regional publication or selection bias.

Similarly, some lncRNAs (e.g., MALAT1, TUG1, lnc-DC) showed subtype-specific expression patterns (SPMS vs. RRMS) in individual studies; however, the data are too sparse and heterogeneous to support robust, generalizable conclusions. Future research should consistently report participants’ ethnicity, geographic origin, and MS subtype to enable meta-regression and formal assessment of geographic and subtype effects. The QUADAS-2 assessment revealed that a minority of studies (*n* = 3) were rated as having a high risk of bias in patient selection, primarily due to non-random or non-consecutive sampling. Additionally, some studies lacked clear reporting on aspects such as assay timing and blinding of index test assessors, contributing to unclear risk in the “Index Test” domain. While QUADAS-2 does not explicitly capture population or recruitment overlap, we noted that a proportion of studies often involved recurring authors and institutions. Although not confirmed, this raises the possibility of shared institutional patient pools, which could lead to redundant sampling across publications and also redundant findings with not real independent replication. While this overlap is speculative, it should be considered when interpreting the overall weight of evidence.

### 4.4. Implications for Future Research

To advance the understanding of lncRNAs in MS, several critical steps are needed. For instance, shifting toward discovery-based rather than hypothesis-driven lncRNA profiling by employing bulk or single-cell RNA sequencing in untargeted ways across diverse MS phenotypes and tissues. Rather than limited cohort availability, the underrepresentation of studies from Europe, North America, and East Asia likely reflects a delayed integration of lncRNA-focused analyses into existing transcriptomic and genetic research programs. As the functional relevance of lncRNAs in MS biology becomes clearer, future investigations can leverage well-established MS consortia and datasets to systematically incorporate lncRNA profiling alongside coding transcript and GWAS-based studies. This could clarify whether lncRNA regulation represents an additional layer of disease heterogeneity beyond known genetic risk loci. Moreover, emerging evidence suggests that lncRNA expression may differ across MS subtypes—some lncRNAs, such as GAS5 and MEG3, appear more consistently dysregulated in RRMS, whereas data for SPMS or PPMS remain sparse. Addressing these subtype-specific differences through longitudinal and multicenter designs could clarify whether certain lncRNAs track with disease activity, progression, or treatment response.

Encouraging collaboration among multiple countries and research centers to validate findings across various ethnic and geographic groups can improve reproducibility and generalizability. Establishing standardization in sample handling (such as PBMCs versus serum), assay platforms (including qRT-PCR and RNA-seq), and clinical classifications (MS subtype, relapse status, treatment history) may address challenges related to heterogeneity that affect comparison and possible meta-analytic efforts. Finally, increasing biological knowledge and characterization of these and other ncRNA species will hopefully refine the regulatory landscape in MS.

### 4.5. Strengths and Limitations of This Review

Strengths of this review include its comprehensive scope, systematic selection of studies, and application of the QUADAS-2 tool for bias assessment. Moreover, by mapping lncRNA expression patterns alongside functional interpretations (Table 2), the review provides a unique synthesis not just of prevalence but of biological context. However, key limitations include the inability to perform meta-analysis due to extreme heterogeneity in study design, assay types, and reporting formats. Additionally, we should also consider publication bias, given that negative or non-significant lncRNA findings are less likely to be reported, potentially skewing the apparent direction of expression. The strong geographic clustering of studies in the MENA region further limits generalizability and highlights the urgent need for broader, discovery-driven, and internationally coordinated lncRNA research in MS.

## 5. Conclusions

This systematic review highlights the increasing attention given to lncRNAs as both biomarkers and modulators in the pathophysiology of MS. Some lncRNAs, such as MALAT1, GAS5, MEG3, and H19, demonstrated consistent expression profiles across multiple studies, with cumulative evidence of their involvement in MS. Others, including TUG1, HOTAIR, THRIL, and IFNG-AS1, displayed more context-dependent patterns influenced by disease stage, relapse/remission status, treatment exposure, sex, or ethnicity. Despite this variability, these lncRNAs converge on central immunological pathways such as NF-κB, IFN-γ/Th1, STAT3, and glucocorticoid signaling, underscoring their biological plausibility. However, progress in this field is currently limited by factors including geographic concentration, preselection bias, and a lack of comprehensive discovery-based research. To facilitate clinical application, future investigations should emphasize multi-center collaborations, the use of standardized methodologies, and the adoption of unbiased transcriptome-wide design. Overcoming these obstacles is critical to fully harness the diagnostic and mechanistic value of lncRNAs in MS.

## Figures and Tables

**Table 1:** A priori defined inclusion and exclusion criteria according to the Population (P), Intervention (I), Comparison (C), Outcomes (O) and Study design (S) (PICOS) framework.

Items	Inclusion Criteria	Exclusion Criteria
Population	Human participants diagnosed with MS, including all clinical subtypes.	Pediatric patients (age at onset <18 years), less than 20 subjects were involved in total in the study.
Intervention	Measurement of lncRNAs in blood or CSF using molecular techniques such as qRT-PCR, RNA sequencing, or microarray profiling.	
Comparator	Healthy individuals or neurological disease controls	
Outcome	Primary outcome: Differential expression of lncRNAs between MS patients and controls. Secondary outcomes (if reported): association of lncRNAs with clinical features, immune/inflammatory markers, or biological pathways.	
Study Type	Peer-reviewed original human studies with observational designs (e.g., case–control, cohort).	Reviews, editorials, conference abstracts, case reports, non-peer-reviewed publications, animal or cell-based studies, and database-only analyses.

**Table 2:** Summary of findings on dysregulation of lncRNAs in MS patients compared with HCs.

lncRNA	Included Studies (*n*)	Direction of Change in MS vs. HC	Consistency	Functional Implications	References
*MALAT1*	5	↑ in 2; ↓ in 1; NS in 2	Partially consistent	Modulates alternative splicing of MS-related genes; its suppression promotes pro-inflammatory macrophage and T-cell phenotypes (anti-inflammatory role suggested)	[15,16,17,18,19]
*GAS5*	4	↑ in all	Consistent	Acts as a decoy glucocorticoid receptor repressor, inversely correlated with NR3C1; may modulate inflammatory responses and glucocorticoid sensitivity	[8,20,21,22]
*THRIL*	3	↑ in all	Consistent	Correlated with TNF-α; implicated in NF-κB innate signaling via hnRNPL-mediated TNF-α induction	[23,24,25]
*MEG3*	3	↓ in all	Consistent	Reduced MEG3 may reflect immune activation via NLRC5 de-repression; acts as tumor suppressor and immune modulator	[26,27,28]
*IFNG-AS1*	4	↓ in 1; ↑ in 3	Partially consistent	Enhances IFN-γ transcription via T-bet and H3K4 methylation; amplifies Th1 responses; may suppress IL-17 and promote immune regulation	[4,29,30,31]
*WFDC21P*	3	↑ in 2; NS in 1	Consistent	It is ↑ in female RRMS, suggesting a gender-dependent role in MS pathogenesis and potential as a biomarker for disease activity; correlates with MALAT1, indicating co-regulation in autoimmune responses.	[18,32,33]
*HOTAIR*	2	↓ in 1; NS in 1	Inconsistent	Pro-inflammatory NF-κB target (↑MMP9); context-dependent regulation	[16,21]
*TUG1*	2	↑ in 1; ↓ in 1	Inconsistent	Context-dependent role in MS pathogenesis, potentially influencing immune regulation; inverse correlation with disease duration in females indicates a possible gender- and stage-specific function.	[17,34]
*H19*	2	↑ in all	Consistent	Correlation with VDR suggests involvement in vitamin D–mediated immune regulation	[7,16]

Note: ↑: upregulated; ↓: downregulated; NS: not significant.

**Table 3:** Findings on lncRNAs according to MS sub-phenotype analyses (treatment, relapse status, clinical course).

lncRNA	Study Context/Comparison	Direction of Change	Functional Implications	References
*THRIL*	MS vs. HC stratified by Iranian ethnicities (Kurdish vs. Sistani)	↑ in Kurdish MS vs. HC (*p* = 0.03); ↓ in Sistani MS vs. HC (*p* < 0.05)	*THRIL,* upstream regulator of STAT3; ethnic-specific differences suggest gene–environment interactions influencing immune dysregulation in MS.	[33]
*MEG3*	Treatment-naïve RRMS vs. RRMS under different DMTs (IFNβ-1a, fingolimod, GA, DMF) and vs. HC	NS overall; ↓ in fingolimod-treated females	MEG3 interacts with T-bet/IFN-γ axis; differential modulation by DMTs highlights drug-specific regulation of immune signaling.	[35]
*HOTAIR*	RRMS relapse vs. remission	↑ in relapse vs. remission	HOTAIR upregulation is positively correlated with TNF-α and MMP9, suggesting a role in promoting inflammatory and tissue remodeling pathways during acute MS relapse.	[24]
*HOTAIR*	RRMS before and after VitD supplementation vs. HC	↑ in VitD-deficient RRMS vs. HC; ↓ after vitamin D supplementation, although not statistically significant	HOTAIR may influence MS via epigenetic regulation and immune/neuroglial modulation; upregulated in VitD-deficient patients, indicating a role in vitamin D–mediated effects	[36]
*TUG1*	SPMS vs. HC	↑ in SPMS vs. HC	Regulation of autoimmune and inflammatory pathways inferred via predicted interaction with miRNAs involved in MS	[37]
*H19*	RRMS relapse vs. remission	↑ in relapse phase; positively correlated with IL-6	Involved in NF-κB–mediated inflammatory responses; correlation with IL-6 links it to pro-inflammatory pathways. Diagnostic potential to distinguish relapse from remission in RRMS.	[24]
*H19*	SPMS vs. RRMS	↑ in SPMS vs. RRMS; higher in females	Elevated levels in SPMS and in females highlight its potential as a biomarker of disease activity, progression, and sex-specific susceptibility	[7]

Note: ↑: upregulated; ↓: downregulated; NS: not significant; DMTs: Disease-Modifying Therapies; IFNβ-1a: Interferon β-1a; GA: Glatiramer Acetate; DMF: Dimethyl Fumarate.

## Data Availability

No new data were created or analyzed in this study.

## Supplementary Materials

The following supporting information can be downloaded at https://www.mdpi.com/article/10.3390/genes16111327/s1, Table S1: List of differentially expressed lncRNAs from the included studies. Table S2: Summary of vote-counting analysis for frequently studied lncRNAs in MS, indicating the number of studies reporting upregulation, downregulation, or no significant difference across biological sample types.

## Abbreviations

LncRNA: long non-coding RNA; MS: Multiple Sclerosis; PPMS: Primary Progressive Multiple Sclerosis; RRMS: Relapsing-Remitting Multiple Sclerosis; SPMS: Secondary Progressive Multiple Sclerosis; PBMC: Peripheral Blood Mononuclear Cell; CSF: Cerebrospinal Fluid; DMTs: Disease-Modifying Therapies; HC: Healthy Controls; HGNC: HUGO Gene Nomenclature Committee; QUADAS-2: Quality Assessment for Diagnostic Accuracy Studies-2; RT-PCR: Real Time Polymerase Chain Reaction
